# Serial Tissue Expansion and Skin Grafts in the Management of a Giant Congenital Nevus of the Face: Review of Literature and Case Report

**DOI:** 10.1055/a-2201-8061

**Published:** 2024-02-29

**Authors:** Trần Thiết Sơn, Phan Tuấn Nghĩa, Phạm Thị Việt Dung, Tạ Thị Hồng Thuý, Hoàng Tuấn Anh, Lê Anh Huy

**Affiliations:** 1 Department of Plastic and Reconstructive Surgery, Hanoi Medical University, Hanoi, Vietnam; 2 Department of Plastic Reconstructive and Aesthetic Surgery, Bach Mai Hospital, Hanoi, Vietnam; 3 Department of Plastic Reconstructive and Aesthetic Surgery, University of Medicine and Pharmacy, Hanoi National University, Hanoi, Vietnam

**Keywords:** giant congenital nevus, tissue expansion, facial nevus, head and neck reconstruction

## Abstract

Giant congenital nevi, especially on the head and neck, pose a challenge for plastic surgeons. This requires extensive experience in detailed planning, combining different techniques, and selecting appropriate materials for reconstruction. There have been reports of using a tissue expander, serial resection method, and full-thickness skin grafts for this type of nevus. However, the best way to completely remove a giant congenital nevus is endless. In this article, we would like to present a case of a left hemifacial giant congenital nevus in which we used multiple tissue expansion to fully replace the nevus, along with some of our modification techniques.

## Introduction

Giant congenital nevi (GCN) are rare neuroectodermal lesions that can occur anywhere on the body. Hemifacial lesions are the most difficult to treat among the head-related GCN. Early surgical management of these lesions is recommended due to their malignant potential. 1 2 The surgical literature documents the use of tissue expansion to repair significant defects resulting from GCN resection on the face. 3 4 Occasionally, serial tissue expansion is necessary to recruit enough skin for complete facial reconstruction. 5 This case demonstrates the successful surgical resurfacing of a hemifacial GNC using serial tissue expansion and full-thickness skin grafting. It highlights several technical points that may facilitate the use of this method.

## Case

**Fig. 1:**
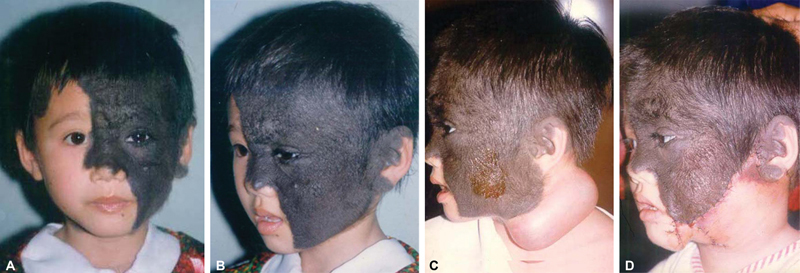
A 4-year-old girl with GCN of the left hemifacial, including the left forehead, nasal bridge, eyelids, cheek, and submandibular region. ( **A** ) Front view. ( **B** ) Lateral view. ( **C** ) The end of the injection of the first expander was placed in the left upper neck. ( **D** ) The lower nevus was excised, and a cervicofacial rotation–advancement flap was used to cover the defect. GCN, giant congenital nevi.

A 4-year-old girl presented to our department with a giant left hemifacial congenital nevus. The affected area included the left forehead, nasal bridge, eyelids, cheek, and submandibular region ( Fig. 1A, B ). Physical examination revealed a 16 × 20 cm (170 cm ^2^ ) black nevus that was hard and firm on palpation. The portion of the nevus extending onto the cheek and the submandibular area was measured at 90 cm ^2^ . During the consultation, the patient's parents refused to use forehead tissue expansion; therefore, we proposed serial tissue expansion for coverage of the left cheek and neck and full-thickness skin grafts for the left forehead and upper eyelid area.

During the first surgery, a 100-mL rectangular tissue expander was inserted beneath the platysma in the left upper neck through an intralesional incision. One week after the operation, the expander was injected with normal saline until the onset of discomfort. Similar subsequent injections were performed at 1- to 2-day intervals. In 5 weeks, the prosthesis was expanded to 150 mL ( Fig. 1C ). Twelve days after the last injection, the expander was removed, the lower portion of the nevus was excised, and the inferiorly based expanded flap was transferred to the defect as a cervicofacial rotation–advancement flap. The part of the capsule that was superficial to the implant was partially excised, removing only the innermost layer of the capsule but leaving the more vascular outer portion undisturbed. Advancement of the expanded skin resurfaced a 4 × 10 cm (∼34 cm ^2^ ) area of the lesion on the cheek ( Fig. 1D ).

**Fig. 2:**
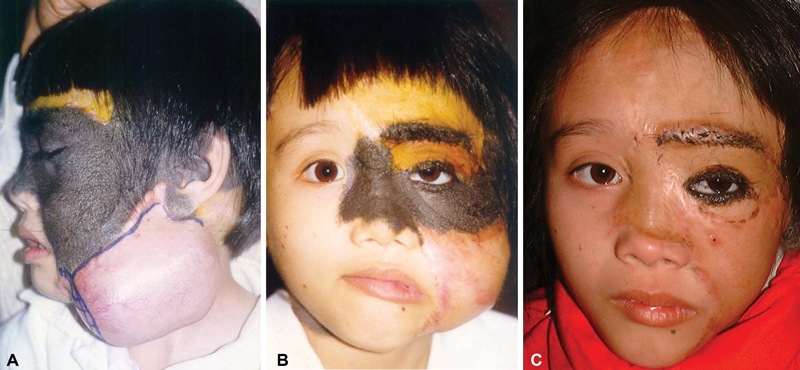
( **A** ) The nevus on the forehead was excised, and a full-thickness skin graft was used to cover this defect. A second expander was placed in the part of the previously expanded skin, and re-expanded skin restored the lower cheek. ( **B** ) The third tissue expander is placed beneath the previously expanded cheek skin. ( **C** ) The third re-expanded flap was advanced to cover the remaining cheek nevus, and the nasal bridge portion of the nevus was replaced with a supraclavicular full-thickness skin graft. One-year follow-up result.

The second stage began 6 months after the expanded skin had regained some elasticity and the scars had matured. A rectangular 150-mL expander was placed in the previously extended lower cheek skin region. At the time of expander placement, the forehead nevus was excised, and a full-thickness skin graft from the abdominal area was used to cover this defect. The expander was inflated to a total volume of 200 mL over 3 weeks ( Fig. 2A ). Five weeks after insertion, the expander was removed, and an additional 4 × 10 cm area (∼30 cm ^2^ ) of the lesion was excised and resurfaced with the re-expanded flap. Most of the patient's cheeks had been replaced with normal skin.

After another 5 months, the third stage began with another rectangular 150-mL expander placed beneath the previously expanded cheek skin. Seven days after insertion, the expansion started gradually using a similar regimen. It was completed at a volume of 180 mL after 4 weeks ( Fig. 2B ). Approximately 6.5 weeks after placement, the expander was removed, and the expanded flap was advanced to complete the resurfacing of the remaining 3 × 10 cm (∼26 cm ^2^ ) of cheek nevus. The flap was sutured to the infraorbital rim to secure its position. At this time, the nevus' nasal bridge and upper eyelid portions were also excised and resurfaced with supraclavicular full-thickness skin grafts.

**Fig. 3:**
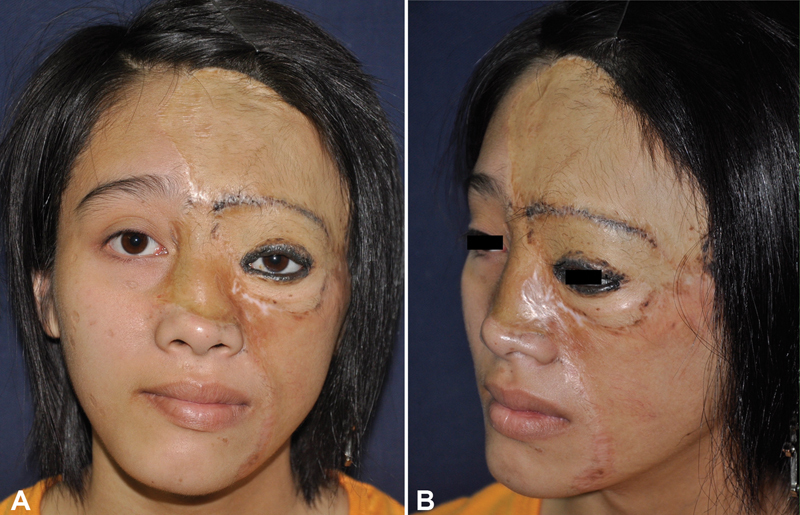
Thirteen years after the last expansion, the scars heal well, and a satisfactory aesthetic result appears to have been obtained. ( **A** ) Front view. ( **B** ) Lateral view.

After completing these three stages, the patient still had 3 mm of residual nevus along the margin of the lower eyelid and 20 cm ^2^ in the left temporal scalp region. Retraction of the cheek flap resulted in a slight ectropion that was treated by release and placement of a full-thickness supraclavicular skin graft that allowed recreation of the lower eyelid margin at the same time ( Fig. 2C ). Thirteen years following the last expansion and the scars have healed well. Satisfactory aesthetic results were acceptable ( Fig. 3 ).

## Discussion

The literature has given multiple criteria to determine the dimensions necessary to define a congenital nevus as a “giant.” 1 Despite this debate, giant pigmented nevi, especially of the face, create major disfigurement and significant psychosocial difficulty for the children and their parents. Cosmetic deformity is usually their primary concern. However, the association of these nevi with the development of malignant melanoma offers an additional reason for excision. For smaller facial lesions, complete nevus removal can be achieved by serial excisions or skin grafting. 6 7 8 9 Autologous skin grafts are often split-thickness, full-thickness, or combined acellular dermal matrix (Intergrat or Matriderm) and split-thickness skin grafts. 10 These techniques are simple and easy to implement for even large facial GCN. However, the main disadvantage is that the cosmetic results are not as desired and unpredictable, the skin grafts are pigmented and hairy, and the thickness of the graft becomes thinner over time. The tissue expansion remains the primary basis for resurfacing larger areas for extensive pigmented nevi. 11 12 The main advantage of tissue expansion is the ability to provide significant amounts of color- and texture-matched skin with potentially minor donor-site morbidity. 13 14 15 Occasionally, in patients with a very large cheek nevus, as seen in the case presented, more than just a single expansion may be needed to resurface the involved area. 16 17 Although an expanded cervicofacial flap could be advanced from the mandibular region upward and medially, it is difficult to reach the medial canthus in one stage. 18 In this scenario, serial expansion may be helpful.

In 1986, Sellers et al presented the first case of using serial tissue expansion to replace a sizeable split-thickness skin graft on the thigh. They noted that the skin can be re-expanded and advanced several times to cover significant defects. This is based on the enhanced capillary network found on the expanded skin. 19 Perlyn et al used serial large volume tissue expansion as a secondary reconstruction for skin graft scar contracture after removing a giant congenital nevus. This method became a salvage as other techniques cannot provide enough material and it was risky to do by a single expansion. 20 Hudson et al operated on patients with giant hairy nevus and burns with two to four times serial expansion. The authors confirmed that re-expansion is a safe and effective procedure for achieving better pediatric plastic surgery results. The paper also mentioned about the thickened capsule at the base of the expander, which limits the flap advancement. 5 Lee et al reviewed serial expansion performed for giant congenital melanocytic nevi in 77 pediatric patients. It was found that a positive correlation between the number of surgeries and the expansion rate; a negative correlation between the large expander size and the expansion rate. That means an increased number of expansions will induce greater flexibility, but the the larger expander will restrict the expansion rate. 21 In 2020, Kim et al reviewed the medical records of 88 patients with tissue expander reconstruction, including 33 patients with serial expansion. By comparing single and serial expansion techniques, Kim et al concluded that infection and revisional operation rates were significantly higher in the serial expansion group than in the single expansion group. He also reported that the large expander insertion size and large inflation volume increased the risk of complications. Serial expansion can provide cosmetic surgical outcomes for pediatric patients with appropriate indications. 16

Our patient's single-stage tissue expansion could not achieve complete coverage because the nevus extended to half of the face. It was not possible to insert two or more expanders into the remaining normal skin on the cheek, so we opted for serial excisions and expansion, which enabled us to resurface the entire nevus (90 cm ^2^ ). Other parts of the face (i.e., nose dorsum, upper eyelid, and left hemiforehead) were resurfaced with full-thickness skin grafts.

This case illustrates several points to consider. The first is that the lateral neck skin can be serially expanded to allow it to reach the lower eyelid. After three full expansions, the re-expanded skin flap maintains its thinness, color, and texture match. There are two different ways to excise all capsular formations around the expander in this patient. We removed the deep (below-the-expander) capsule entirely. Regardless, the superficial (above-the-expander) capsule was only partially excised. Only the inner layer adjacent to the expander was removed, leaving the more vascularized outer layer beneath the flap intact. This maneuver created a raw surface under the flap that was less prone to seroma formation than a capsule. At the same time, it enables the flap to be advanced more and minimal obscuring of the underlying facial features. A robust blood supply is preserved by leaving the outer vascular portion of the capsule intact to the flap. Thus, the combination of subtotal capsulectomies and rotation–advancement of the expanded flaps maintains the blood supply while avoiding much of the downward contraction seen with these flaps. We waited 5 to 6 months between expansions to allow the flap to heal and for skin laxity to recover. Then another expander was inserted under the previously expanded flap, and further expansion began. In subsequent surgeries, mild fibrosis was observed beneath the flap, and this dissection proceeded quickly. The second point demonstrated by this case is that we were able to perform multiple expansions in the facial region without significant problems. This also confirms the findings of other authors, who have reported relatively low complication rates of 6 to 9%. We use tissue expansion regularly in our department and have made several modifications to our technique to minimize our risk of further complications. We have modified our techniques to begin expansion earlier and accelerate the expansion process because many of our patients come from remote rural communities and have to stay in the hospital for the entire treatment period. Instead of placing the expander through a border or distant incision, all expanders are put through an intralesional incision. A tunnel is made beneath the nevus to place the implant far from the incision under the adjacent normal skin. A three-layer closure with nonabsorbable sutures provides and maintains incisional strength. In addition, the undermined area between the expander and the incision was re-approximated with sutures from the platysma to the underlying tissue. These sutures reduce the tension transfer from the expansion site to the incision. Expansion is typically started at the end of the first week. In addition, during the first 2 weeks after expander placement, we try to inject as often as possible, approximately four times a week, because the absence of a capsule makes the skin less resistant to expansion. This suturing technique has been successfully applied to our patients indicated for reconstruction with a tissue expander. 22

### Conclusion

We report a patient with a hemifacial giant congenital nevus. Using serial tissue expansion, we replaced approximately 90 cm ^2^ of the lesion with neck skin. This case demonstrates serial tissue expansion's value during resurfacing of a giant congenital nevus of the face. In addition, using intralesional incisions for expander placement, separation of the implant pocket from the incision with sutures, and peri-implant seroma aspiration may facilitate earlier and more rapid expansion while minimizing the risk of complications.
